# Cognitive functioning and soccer heading: one-year longitudinal assessment among professional players

**DOI:** 10.1055/s-0045-1805051

**Published:** 2025-03-19

**Authors:** Giovanni Batista Palma, Mariana Drummond Martins Lima, Ana Carolina Oliveira Rodrigues, Clarisse Vasconcelos Friedlaender, Celso Furtado de Azevedo Filho, Rodrigo Campos Pace Lasmar, Paulo Caramelli

**Affiliations:** 1 Universidade Federal de Minas Gerais, Faculdade de Medicina, Grupo de Pesquisa em Neurologia Cognitiva e do Comportamento, Belo Horizonte MG, Brazil.; 2 Universidade Federal de Minas Gerais, Faculdade de Medicina, Programa de Pós-Graduação em Ciências Aplicadas à Saúde do Adulto, Belo Horizonte MG, Brazil.; 3 América Futebol Clube, Departamento Médico, Belo Horizonte MG, Brazil.; 4 Clube Atlético Mineiro, Departamento Médico, Belo Horizonte MG, Brazil.; 5 Faculdade de Ciências Médicas de Minas Gerais, Belo Horizonte MG, Brazil.

**Keywords:** Cognition, Neurology, Athletes, Soccer, Brain Injuries, Traumatic

## Abstract

**Background** Soccer is the most popular sport worldwide, and the only one in which players purposely and repetitively use their head to hit the ball. In recent years, more attention has been given to subconcussive impacts, which are characterized by a cranial impact that does not result in known or diagnosed concussion on clinical grounds.

**Objective** To investigate the effects of soccer heading on cognitive functioning among professional soccer players.

**Methods** In a longitudinal case-control study, 22 professional soccer players were compared with 37 non-athletes on 2 occasions (T0 and T1) separated by a 1-year interval. The cognitive assessment consisted of computerized neuropsychological tests and the Brief Neuropsychological Assessment Battery (NEUPSILIN).

**Results** There was no evidence of cognitive impairment among athletes, who actually outperformed the controls in terms of score, accuracy, and reaction time in certain tests. Moreover, the estimates of heading exposure did not correlate with the cognitive performance of players. The intragroup analyses revealed that while the controls improved their performance on three cognitive variables from T0 to T1, no improvement occurred among players. However, within-group variation in performance from T0 to T1 was similar.

**Conclusion** Although the present study has not shown an association between soccer heading and cognitive impairment, subconcussive impacts may have a negative effect on brain function, as improvement in cognitive performance was not observed among athletes. Future and longer longitudinal investigations are needed to clarify the relationship between soccer heading and cognition.

## INTRODUCTION

Since the twentieth century, the role of concussion in chronic neurodegenerative disease has gained attention, while athletes have shown neuropsychiatric symptoms and cognitive impairment after numerous head impacts throughout their competitive years. Nowadays, those repeated brain injuries are known as a potential cause of chronic traumatic encephalopathy (CTE). 1

In recent years, more attention has been given to subconcussive impacts. The term “subconcussive” refers to head impacts that could lead to neuronal dysfunction without causing the typical concussive symptoms of confusion, amnesia, dizziness, visual disturbances, or headaches. 2 Moreover, the cumulative effects of repetitive minor injury may not manifest for many years, as in CTE. 3 Therefore, pathological evidence of traumatic brain injury is mixed, and, if detectable, it is likely to present prior to the onset of overt symptoms or disability. 4 5

Soccer is the most popular sport worldwide, and the only one in which players purposely and repetitively use their head to hit the ball, on an average of 6 to 12 headings per competitive game, in which the ball reaches high velocities. 6 Therefore, even though concussive events are not common in soccer, we can consider that headings may act as subconcussive impacts which, while not causing immediate symptoms, may contribute to long-term cognitive damage. Moreover, based on field position and career length, professional soccer players are exposed to a higher risk of developing neurodegenerative disease, and a more frequent use of dementia-related medications. 7 8 9 Importantly, the possible negative effects of headings may depend on the rate of exposure, the time between exposures, and the vulnerability of individual players. 3

However, to date, research on the possible effects of soccer headings on brain function has produced controversial results, with some studies 10 11 12 suggesting an association between heading and cognitive impairment, while others 13 14 15 have found no correlation.

The current one-year longitudinal case-control study aims to investigate the effects of soccer headings on cognitive functioning among professional soccer players. We have previously reported the baseline and cross-sectional results of the study, 16 in which no evidence of cognitive impairment was found among soccer players compared with non-athletes, and no association between heading exposure and neuropsychological performance was observed.

## METHODS

Two groups of male participants matched by age and years of schooling participated in the study: 22 active professional soccer players (mean age = 24.1 ± 5.2 years; mean years of schooling = 11.2 ± 1.7 years) from 2 teams playing in the Brazilian A-series championship. We also included 37 non-athletes as controls (mean age = 26.1 ± 4.0 years; mean years of schooling = 10.9 ± 1.7 years).

The athlete's group was composed of 5 goalkeepers, 7 defenders, 7 midfielders and 3 forwards. All athletes performed approximately 15 hours of training sessions per week and usually played one or, during most of the soccer season, two competitive matches per week.

The control group was composed of non-athletes who worked as doormen or security guards and who did not practice soccer or practiced it occasionally and at a recreational level.

To characterize the participants, a sociodemographic questionnaire was applied, as well as parts of the Mini International Neuropsychiatric Interview (MINI), 17 with the aim of investigating possible psychiatric disorders. None of the participants showed a major depressive episode or alcohol dependence, nor reported using any psychotropic drug.

The soccer players were also questioned about the average number of headings per game. In the cross-sectional analysis of the project of the present research, 16 the number of headings performed by 16 athletes in 42 matches was directly measured by an observer. Considering this subsample of players, a comparison between the self-reported and the actual number of headings per game revealed no significant differences. Moreover, a significant correlation was found between the self-reported and the actual values. 16 Therefore, we believe that the self-report method used in the current study may be considered a good estimate of headings during official matches.

In relation to previous reports of concussion, 7 players (31.8%) and 4 controls (10.8%) reported history of concussive impacts ( *p* = 0.045).

A total of six professional players reported regular alcohol use, but none used cigarettes or other toxic substances. Only one player was on medication affecting the central nervous system (escitalopram), none had a history of major depressive episodes, and one soccer player had generalized anxiety disorder. Among the controls, no participant reported using drugs with effects on the central nervous system, nor exhibited mood disorder or alcohol dependence. Importantly, no participants from the 2 groups had consumed alcohol in the 12 hours prior to the neurocognitive tests, and only 1 had consumed alcohol in the 24 hours leading up to the tests.

The participants were tested on two occasions, T0 and T1, separated by a 1-year interval. In T0, 7 players and 4 non-athletes referred to a lifetime history of concussion, with a significant difference in the rate of concussed subjects between groups ( *p* = 0.045). No concussion was reported between T0 and T1 analysis. On both occasions, the cognitive assessment consisted of 2 parts: the first part lasted ∼ 35 minutes and was composed of computerized neuropsychological tests designed on the E-Prime (Psychology Software Tools, Inc., Pittsburgh, PA, United States) software, 18 which measure general motor skills, memory, attention, and executive functioning. On each test, the reaction time in milliseconds and the percentage of correct responses were calculated.

### Computerized tests

- Simple Reaction Time Test: the participants press the “1” key in response to a “*” symbol appearing on the screen at random intervals. The test contains 40 trials in a single block.
- Immediate Memory Test: the participants memorize 20 words shown on the screen and then identify them from sets of 4 words. The test contains 3 blocks of 20 trials.
- Attention Test: the participants determine whether a target figure appears among four similar figures. The test includes 60 trials in a single block.
- Number-Letter test: the participants categorize numbers as odd/even or letters as consonants/vowels depending on their screen position. The test involves task switching with 32 trials in 2 blocks and 64 in a third block.
- Two-Back test: the participants monitor sequences of numbers, pressing “Enter” when a number matches one from two trials earlier. The test contains 160 trials.
- Stroop test: the participants identify the ink color of incongruent/congruent words, with 72 trials in a single block.
- Delayed memory test: ∼ 30 minutes after the Immediate Memory Test, the participants indicated whether 40 words were in the original list.

The second part of the cognitive assessment was applied immediately after the first one, and it involved the Brief Neuropsychological Assessment Battery (NEUPSILIN), 19 which is composed of 32 short tasks that evaluate different cognitive domains such as time and spatial orientation, attention, perception, memory, arithmetic abilities, language, praxis, problem solving, and verbal fluency. This part also lasted ∼ 35 minutes, and the measured variable was the score on each test.

The statistical analyses were performed using the IBM SPSS Statistics for Windows (IBM Corp., Armonk, NY, United States) software, version 19.0. The normality of each variable was tested with the Kolmogorov-Smirnov test. For independent samples, mean comparisons between groups were performed using the Student's *t* -test for variables with normal distribution, and the Mann-Whitney U-Test, for non-normally distributed variables. Regarding the dependent samples, the paired Student's *t* -test was used for variables with normal distribution, and the Wilcoxon test, for those with non-normal distribution. The correlations involving the athletes' cognitive performance in T0 and T1 and the self-reported number of headings were calculated with the Pearson's Correlation Test, controlling for lifetime history of concussion. The categorical variables were compared using the Chi-squared test. Bonferroni correction ( *p* < 0.01) was used in the present study to enable multiple comparisons involving cognitive performance in T0 and T1. In all other analyses, the significance level was set to 5% ( *p* < 0.05).

All subjects provided written informed consent to participate in the study, which was approved by the Ethics Committee of the Universidade Federal de Minas Gerais (under CAAE 51433115.1.0000.5149).

## RESULTS

In T0 ( Table 1 ), the players performed better than the controls in three variables of the computerized testing, namely reaction time and accuracy in the number-letter test and accuracy in the two-back test. They also outperformed the controls in terms of memory and arithmetic abilities in the NEUPSILIN.

**Table 1:** Cognitive performance of soccer players and controls at T0 and T1

Test	Variable	T0			T1		
		Players	Controls	*p*	Players	Controls	*p*
**Age**	Years	24.13 ± 5.29	26.13 ± 4.09	0.110	25.32 ± 5.38	27.19 ± 4.09	0.137
**Schooling**	Years	11.22 ± 1.75	10.94 ± 1.74	0.888	11.34 ± 1.77	11.08 ± 1.87	0.816
**Computerized tests**							
**Simple reaction time**	Reaction time	290 ± 28	309 ± 39	0.047	280 ± 21	316 ± 37	**0.000**
	Accuracy	100.00 ± 0.00	99.96 ± 0.20	0.441*	100 ± 0.00	100 ± 0.00	−
**Attention**	Reaction time	2,364 ± 580	2,725 ± 679	0.042	2,323 ± 526	2,504 ± 579	0.235
	Accuracy	94.39 ± 4.22	94.09 ± 5.23	0.823	95.83 ± 2.94	95.08 ± 3.38	0.396
** Immediate memory ^a^**	Reaction time	2,377 ± 565	2,935 ± 917	0.013	2,453 ± 492	2,736 ± 768	0.128
	Accuracy	89.39 ± 7.17	86.71 ± 10.55	0.296	91.36 ± 5.26	88.56 ± 8.03	0.150
** Number-letter ^a^**	Reaction time	494 ± 137	656 ± 231	**0.004**	481 ± 116	628 ± 197	0.056
	Accuracy	−1.35 ± 2.78	−5.02 ± 6.76	**0.005**	−0.28 ± 2.14	−1.87 ± 4.10	**0.001**
**Two-back**	Reaction time	518 ± 103	561 ± 100	0.126	503 ± 115	564 ± 129	0.074
	Accuracy	98.66 ± 1.48	94.60 ± 4.95	**0.000**	98.92 ± 1.51	96.37 ± 3.76	0.000*
** Stroop ^a^**	Reaction time	172 ± 97	163 ± 142	0.784	115 ± 138	126 ± 119	0.731
	Accuracy	−2.14 ± 2.96	−1.95 ± 2.92	0.842*	−0.76 ± 3.34	−1.43 ± 3.18	0.842*
**Delayed memory**	Reaction time	874 ± 147	1,049 ± 318	0.019	2,453 ± 492	2,736 ± 768	0.094
	Accuracy	93.75 ± 5.16	92.16 ± 7.68	0.394	91.36 ± 5.26	88.56 ± 8.03	0.617*
**NEUPSILIN battery**							
**Time and spatial orientation**	Score (Total: 8)	8.00 ± 0.00	7.97 ± 0.16	0.441*	7.77 ± 0.43	7.97 ± 0.16	0.441*
**Attention**	Score (Total: 27)	24.31 ± 2.12	23.13 ± 1.90	0.031	24.27 ± 1.69	23.76 ± 1.85	0.056*
**Perception**	Score (Total: 12)	11.00 ± 1.11	11.08 ± 0.92	1.000*	11.41 ± 0.79	11.22 ± 0.75	1.000*
**Memory**	Score (Total: 84)	61.72 ± 6.18	56.29 ± 7.95	**0.008**	64.09 ± 7.07	60.05 ± 8.28	0.061
**Arithmetic skills**	Score (total: 8)	7.59 ± 0.73	6.18 ± 1.94	0.002*	7.63 ± 0.73	6.40 ± 2.06	0.002*
**Language**	Score (total: 53)	50.72 ± 1.69	49.91 ± 2.34	0.164	51.31 ± 1.13	50.43 ± 1.74	0.037
**Praxis**	Score (total: 22)	18.72 ± 1.77	17.62 ± 2.13	0.046	19.18 ± 1.59	18.35 ± 2.08	0.113
**Problem-Solving**	Score (total: 2)	1.81 ± 0.39	1.56 ± 0.55	0.073*	1.86 ± 0.35	1.51 ± 0.55	0.073*
**Verbal fluency**	Score (total: 11)	5.36 ± 1.49	5.08 ± 1.21	0.431	5.77 ± 1.44	5.14 ± 1.39	0.515*

Abbreviation: NEUPSILIN, Brief Neuropsychological Assessment Battery.

Notes: The *p* -values refer to the Student's *t* -test for independent samples, except those marked with the asterisk (*), which refer to the Mann-Whitney's U-test. Bold *p* -values indicate significant differences at a 1% level. In the computerized testing, the variables measured are the mean reaction time, expressed in milliseconds, and the mean accuracy, expressed in the percentage of correct answers. In the NEUPSILIN, the variable measured is the mean score on each test. ªIn the immediate memory, number-letter, and Stroop tests, reaction time and accuracy are calculated as described in the “Methods” section.

In T1 ( Table 1 ), the players outperformed the controls in three variables of the computerized testing: reaction time in the general motor skills test and accuracy in the number-letter and two-back tests. They also showed a better performance in the arithmetic abilities test from the NEUPSILIN.

Intragroup analyses ( Table 2 ) revealed that, while the controls improved their performance in three variables from T0 to T1 (reaction time in the Attention Test and accuracy in the two-back test, and score on the NEUPSILIN memory test), no improvement was observed among soccer players. However, a comparison involving variation in performance between T0 and T1 showed no significant differences between the groups.

**Table 2:** Variation in cognitive performance of soccer players and controls between T0 and T1

Test	Variable	Players			Controls			T1-T0		
		T0	T1	*p*	T0	T1	*p*	Players	Controls	*p*
**Computerized tests**										
**Simple reaction time**	Reaction time	290 ± 28	280 ± 21	0.025	309 ± 39	316 ± 37	0.213	−9.64 ± 18.72	6.65 ± 31.86	0.033
	Accuracy	100.00 ± 0.00	100.00 ± 0.00	−	99.96 ± 0.20	100.00 ± 0.00	0.317*	0.00 ± 0.00	0.03 ± 0.20	0.441*
**Attention**	Reaction time	2364 ± 580	2323 ± 526	0.687	2725 ± 679	2504 ± 579	**0.003**	−41.18 ± 472.06	−220.57 ± 413.64	0.132
	Accuracy	94.39 ± 4.22	95.83 ± 2.94	0.078	94.09 ± 5.23	95.08 ± 3.37	0.256	1.43 ± 3.64	0.99 ± 5.21	0.725
** Immediate memory ^a^**	Reaction time	2377 ± 565	2453 ± 492	0.546	2935 ± 917	2736 ± 768	0.030	76.36 ± 583.28	−199.30 ± 534.69	0.069
	Accuracy	89.39 ± 7.17	91.35 ± 5.26	0.176	86.71 ± 10.55	88.55 ± 8.03	0.172	1.96 ± 6.58	1.84 ± 8.04	0.952
** Number-letter ^a^**	Reaction time	494 ± 137	481 ± 116	0.608	656 ± 231	628 ± 197	0.406	−13.55 ± 121.85	−27.43 ± 198.39	0.740
	Accuracy	−1.35 ± 2.78	−0.28 ± 2.14	0.239	−5.02 ± 6.76	−1.87 ± 4.10	0.013	1.06 ± 4.13	3.15 ± 7.32	0.226
**Two-back**	Reaction time	518 ± 103	503 ± 115	0.352	561 ± 100	564 ± 129	0.783	−15.14 ± 74.55	3.32 ± 72.72	0.354
	Accuracy	98.66 ± 1.48	98.91 ± 1.50	0.511*	94.60 ± 4.95	96.36 ± 3.75	**0.003**	0.25 ± 1.67	1.75 ± 3.39	0.058
** Stroop ^a^**	Reaction time	175 ± 97	115 ± 138	0.053	163 ± 142	126 ± 119	0.120	−57.41 ± 131.47	−36.16 ± 138.20	0.563
	Accuracy	−2.14 ± 2.96	−0.75 ± 3.34	0.155*	−1.95 ± 2.92	−1.42 ± 3.18	0.420*	1.39 ± 4.26	0.52 ± 4.13	0.446
**Delayed memory**	Reaction time	874 ± 147	888 ± 170	0.605	1049 ± 318	990 ± 249	0.057	13.82 ± 123.38	−58.14 ± 179.57	0.103
	Accuracy	93.75 ± 5.16	96.47 ± 3.59	0.029	92.16 ± 7.68	92.97 ± 7.72	0.486*	2.72 ± 5.45	0.81 ± 7.04	0.278
**NEUPSILIN**										
**Time and spatial orientation**	Score (total: 8)	8.00 ± 0.00	7.77 ± 0.42	0.025 *	7.97 ± 0.16	7.97 ± 0.16	1.000*	−0.22 ± 0.42	0.00 ± 0.23	0.012 *
**Attention**	Score (total: 27)	24.31 ± 2.12	24.27 ± 1.69	0.935	23.13 ± 1.90	23.75 ± 1.84	0.167*	−0.04 ± 2.57	0.72 ± 2.34	0.315*
**Perception**	Score (total: 12)	11.00 ± 1.11	11.40 ± 0.79	0.117*	11.08 ± 0.92	11.21 ± 0.75	0.415*	0.40 ± 1.25	0.18 ± 1.04	0.290*
**Memory**	Score (total: 84)	61.72 ± 6.18	64.09 ± 7.07	0.069	56.29 ± 7.95	60.05 ± 8.27	**0.002**	2.36 ± 5.78	3.89 ± 6.89	0.387
**Arithmetic skills**	Score (total: 8)	7.59 ± 0.73	7.63 ± 0.72	0.860*	6.18 ± 1.94	6.40 ± 2.06	0.471*	0.04 ± 0.78	0.27 ± 1.78	0.717*
**Language**	Score (total: 53)	50.72 ± 1.69	51.31 ± 1.12	0.034	49.91 ± 2.34	50.43 ± 1.74	0.100	0.59 ± 1.22	0.59 ± 1.83	0.993
**Praxis**	Score (total: 22)	18.72 ± 1.77	19.18 ± 1.59	0.226	17.62 ± 2.13	18.35 ± 2.08	0.011	0.45 ± 1.71	0.94 ± 1.91	0.326
**Problem-Solving**	Score (total: 2)	1.81 ± 0.39	1.86 ± 0.35	0.655*	1.56 ± 0.55	1.51 ± 0.55	0.414*	0.04 ± 0.48	−0.05 ± 0.40	0.399*
**Verbal fluency**	Score (total: 11)	5.36 ± 1.49	5.77 ± 1.44	0.215	5.08 ± 1.21	5.13 ± 1.39	0.777	0.40 ± 1.50	0.08 ± 1.21	0.362

Abbreviation: NEUPSILIN, Brief Neuropsychological Assessment Battery.

Notes: Soccer players and controls at T0 and T1: the *p* -values refer to the paired *t* -test, except those marked with the asterisk (*), which refer to the Wilcoxon test. Soccer players and controls at T1-T0: the *p* -values refer to the Student's *t* -test for independent samples, except those marked with the asterisk (*), which refer to the Mann-Whitney's U-test. Bold *p* -values indicate significant differences at a 1% level. In the computerized testing, the variables measured are the mean reaction time, expressed in milliseconds, and the mean accuracy, expressed in the percentage of correct answers. In the NEUPSILIN, the variable measured is the mean score on each test. ªIn the immediate memory, number-letter, and Stroop tests, reaction time and accuracy are calculated as described in the “Methods” section.

Among soccer players ( Table 3 ), no significant correlations were found between the self-reported number of headings per game and cognitive performance in T0 nor T1, controlling for concussion history.

**Table 3:** Correlations between cognitive performance of soccer players and self-report of headings per game

Test	Variable	Correlation coefficient (T0)	*p*	Correlation coefficient (T1)	*p*
**Computerized tests**					
** Simple reaction time ^a^**	Reaction time	0.005	0.983	0.039	0.868
	Accuracy	−	−	−	−
**Attention**	Reaction time	−0.398	0.074	−0.188	0.415
	Accuracy	0.178	0.439	−0.130	0.574
** Immediate memory ^b^**	Reaction time	−0.208	0.366	−0.264	0.247
	Accuracy	0.138	0.551	0.194	0.401
** Number-letter ^b^**	Reaction time	0.144	0.533	−0.156	0.500
	Accuracy	−0.217	0.345	0.327	0.148
**Two-back**	Reaction time	0.125	0.589	−0.160	0.488
	Accuracy	−0.006	0.981	0.049	0.832
** Stroop ^b^**	Reaction time	−0.160	0.489	0.084	0.718
	Accuracy	0.288	0.205	0.243	0.287
**Delayed memory**	Reaction time	−0.065	0.780	−0.298	0.189
	Accuracy	0.105	0.650	−0.081	0.726
**NEUPSILIN battery**					
** Time and spatial orientation ^a^**	Score	−	−	0.085	0.714
**Attention**	Score	−0.409	0.066	0.133	0.566
**Perception**	Score	0.179	0.437	−0.106	0.648
**Memory**	Score	−0.263	0.250	−0.063	0.785
**Arithmetic skills**	Score	0.103	0.657	0.405	0.069
**Language**	Score	0.059	0.800	0.208	0.367
**Praxis**	Score	0.228	0.321	0.117	0.613
**Problem-Solving**	Score	0.320	0.157	0.172	0.456
**Verbal fluency**	Score	−0.112	0.628	0.105	0.652

Abbreviation: NEUPSILIN, Brief Neuropsychological Assessment Battery.

Notes: The correlations were measured through the Pearson's correlation test, controlling for the variable “lifetime history of concussion.” ^a^ Correlation tests were not performed for accuracy in the simple reaction time test at T0 and T1 and for score in the time and spatial orientation test at T0, since all subjects had 100% of correct responses and achieved the maximum score respectively. ^b^ In the immediate memory, number-letter, and Stroop tests, reaction time and accuracy are calculated as described in the “Methods” section.

## DISCUSSION

The inconclusive findings on the effects of headings on cognitive impairment among soccer players underscore the need for more robust data on this topic. 20 In the present study, professional soccer players performed better than controls in neuropsychological tests that require task-switching and working-memory abilities, as well as in general motor skills, memory, and arithmetic skills. We believe that this may be related to team sport practice, which demands strategic decision-making in complex and quickly changing contexts. Besides, there is evidence that participation in sports relates to faster processing speed in measures of simple reaction time. 21 This can explain the advantage of professional soccer players in reaction-time measures in T0 (number-letter test) and T1 (general motor skills test).

The present study shows no evidence of impairment in the cognitive functioning of professional soccer players. In fact, athletes displayed shorter reaction times and greater accuracy in some computerized tests, as well as better results in memory and arithmetic skills of the NEUPSILIN. This may be explained by the improvement in motor skills and ability to develop a strategy that comes from sports practice. However, the longitudinal analysis of the participants draws attention. Although there was no setback in the athletes' performance, they showed a less expressive learning curve than the control group.

The relationship between soccer practice and brain function remains controversial, and further studies involving larger samples and longer follow-up are needed to achieve a better understanding of the effects of subconcussive impacts on brain function.

One strength of the present study is the participation of active high-level professional soccer players, since most studies addressing the effects of soccer headings on cognition have not investigated this population. Thus, in the current study, we analyzed individuals who are constantly exposed to many head impacts.

Nevertheless, we must acknowledge that the present study has limitations, such as the fact that soccer players were compared with non-athletes, which is not an ideal comparison group, given the well-established cognitive benefits of physical activity. 22 Hence, physical activity could act as a confounding variable and compromise the study analysis. Moreover, only headings during competitive matches were considered in the study, excluding the training sessions, and possibly underestimating the number of subconcussive impacts. This may have had an influence on our results, which showed no significant differences between soccer players who reported a greater or smaller number of headings per game. Another limitation is that the number of headings was estimated through self-report by the soccer players, which is potentially biased or imprecise. History of concussion was also more frequent among players than controls. Finally, the sample size was small, but this was related to the number of players who agreed to participate and who were available for both the baseline and the one-year evaluations.

In conclusion, the soccer players evaluated in the present study did not show signs of cognitive impairment and actually outperformed the controls in certain tests. However, given the body of evidence linking professional soccer activity with later-life dementia, efforts to optimize head trauma prevention for soccer players need to be considered to reduce long-term risks. 23
